# Construction Notes

**Published:** 1893-06-03

**Authors:** 


### CONSTRUCTION NOTES.

Huddersfield Infirmary.—The Board of the Huddersfield Infirmary is considering how best to increase the accommodation for children, who, until quite recently, were placed in a ward of twelve cots, the front of the ward being enshrouded by the massive stone portico and pillars of the imposing entrance to the Infirmary. As a consequence no sunshine ever reached the ward, and what should have been the brightest ward in the house was dark and dim. In the meantime a small male medical ward has been turned into a children's ward. The old children's ward is used for chronic adult male cases, and the Board is considering the best plan of increasing the accommodation of the Infirmary. The extension will take the form of a new wing, or of an additional storey upon the north wing. The Infirmary has a building fund of over £10,000 already in hand.

